# Importance of Translational Entropy of Water in Biological Self-Assembly Processes like Protein Folding

**DOI:** 10.3390/ijms10031064

**Published:** 2009-03-11

**Authors:** Masahiro Kinoshita

**Affiliations:** Institute of Advanced Energy, Kyoto University/Uji, Kyoto 611-0011, Japan; E-Mail: kinoshit@iae.kyoto-u.ac.jp; Tel. +81-774-38-3503; Fax: +81-774-38-3508

**Keywords:** Protein folding, self-assembly, water, translational entropy, pressure denaturation, cold denaturation, native structure

## Abstract

We briefly review our studies on the folding/unfolding mechanisms of proteins. In biological self-assembly processes such as protein folding, the number of accessible translational configurations of water in the system increases greatly, leading to a large gain in the water entropy. The usual view looking at only the water in the close vicinity of the protein surface is capable of elucidating neither the large entropic gain upon apoplastocyanin folding, which has recently been found in a novel experimental study, nor the pressure and cold denaturation. With the emphasis on the translational entropy of water, we are presently constructing a reliable method for predicting the native structure of a protein from its amino-acid sequence.

## 1. Introduction

Protein folding is the most fundamental and universal example of biological self-assembly, and investigating this process can provide new physical insight into related processes as well. Despite the enormous amount of effort devoted to its study, however, the nature of the folding/unfolding mechanisms has not yet been established. The prediction of the native structure of a protein from its amino-acid sequence, which is one of the most important tasks in modern science and technology, cannot become feasible without first elucidating the mechanisms. It appears that a breakthrough is not likely to be obtained unless a novel concept, which is different from the current approaches, is employed.

Recently, progress in the field of integral equation theories, elaborate statistical-mechanical theories for solvation thermodynamics, has been remarkable. Above all, there is a reliable theory which allows us to calculate the thermodynamic quantities of hydration of a solute, as well as the density and orientational structures of water near the solute. In the theory water is not a dielectric continuum but an ensemble of water molecules. The number of water molecules is infinitely large, and even large polyatomic solutes like proteins can be treated as described in later sections. Moreover, the thermodynamic quantities of hydration can be decomposed into various components which provide physical insight. This progress has shed light on some important problems related to biological self-assembly processes like protein folding. We focus this review on the new results or concepts thus obtained.

We have pointed out that upon protein folding, the restriction of the translational movement of water molecules (i.e., water crowding) in the system is remarkably reduced, leading to a large gain of the water entropy [1,2]. This water-entropy gain is a principal driving force in a variety of self-assembly and ordering processes in biological systems [3–7] (e.g., protein folding, molecular recognition, and association of protein molecules). According to the usual view [8,9], the water adjacent to a nonpolar group is entropically unstable, and protein folding is driven by the release of such unfavorable water to the bulk through the burial of nonpolar side chains. Thus, even in the usual view a water-entropy gain occurring upon the folding is regarded as a significant driving factor. However, only the water in the close vicinity of the protein surface is taken into consideration and the water-entropy effect is argued mainly in terms of the protein-water orientational correlation, enhanced hydrogen-bonding of the water network, and restriction of the rotational freedom of water molecules. In this article, we briefly review our studies on the folding/unfolding mechanisms of proteins with the emphasis on the effect arising from the translational movement of water molecules. By comparing our theoretical results with recently reported thermodynamic data for apoplastocyanin folding, we demonstrate that the folding actually accompanies a large gain in the water entropy, which dominates the loss of the conformational (intramolecular) entropy of the protein molecule [10]. Pressure and cold denaturations are shown to be striking examples which cannot be rationalized by the usual view (i.e., the view looking only at the water in the close vicinity of the protein surface [11–15]). We also describe our ongoing efforts toward the development of a reliable method for predicting the native structure of a protein [16,17], which is based on our unique view (readers should refer to our earlier cited publications for more details).

## 2. Translational-Entropy Gain of Water upon Protein Folding

**Figure 1.**
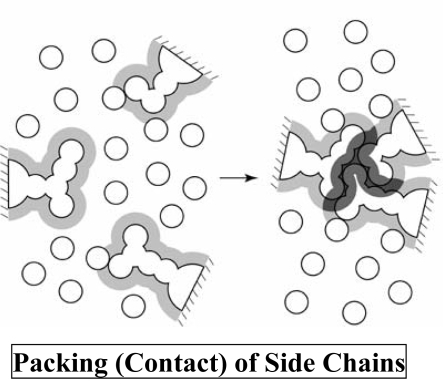
The presence of a side chain generates an excluded volume (EV) which the centers of water molecules cannot enter. The EV is the volume occupied by the side chain itself plus the volume shown in gray. When side chains are closely packed (or contact one another), the EVs overlap and the total volume available to the translational movement of water molecules in the system increases by the overlapped volume shown in black, leading to a gain of the water entropy. It has been argued for a solute with cylindrical shape generating an EV that the formation of the helical structure by a cylinder or the lateral contact of cylinders leads to a reduction of the EV [6,7]. This argument can be applied to the backbone of a protein. The construction of the α-helix accompanies not only a formation of the helical structure by a portion of the backbone but also the contact of side chains. The lateral contact of portions of the backbone and the contact of side chains occur in the construction of the β-sheet. Thus, the construction of these secondary structures leads to a reduction of the EV followed by a corresponding gain of the water entropy [1,2].

We have shown that even in the complete absence of potential energies among the atoms in a protein-aqueous solution system, there is a physical factor which strongly favors the folded state of the protein [1,2]: the translational movement of water molecules. A simple illustration based on the Asakura-Oosawa (AO) theory [18,19] is shown in Figure 1. The AO theory often becomes problematic as described in 2.2, 2.4, and 3.1, but its simplicity is convenient to provide a rough explanation of the following excluded volume (EV) effect. Upon protein folding, the EV for water molecules in the system is largely decreased (here, the EV is defined as the volume of the space which the centers of water molecules cannot enter). As a consequence, the total volume available to the translational movement of water molecules is correspondingly increased, leading to a large gain of the water entropy. Within the framework of the AO theory, the entropic gain is given by −*k*_B_*N*_S_*ΔV*_ex_/*V*=−*k*_B_*ρ*_S_*ΔV*_ex_ where *k*_B_ is the Boltzmann constant, *N_S_* the total number of water molecules in the system, *V* the system volume, *ΔV*_ex_ (<0) the decrease in the EV, and *ρ*_S_=*N*_S_/*V*. By “translational movement of water molecules” we do not refer to dynamical aspects of the behavior of the water-protein system. What we claim is that upon the folding the number of accessible translational configurations of water in the system increases to a large extent.

In our earlier work [1,2], a protein was treated as a set of fused hard spheres (the details of the complex polyatomic structure are fully taken into consideration) immersed in a hard-sphere solvent (its number density and particle diameter were set at the values for water). In this model system, all the allowed configurations share the same energy and the system behavior is purely entropic in origin. The three-dimensional integral equation theory [2,4,6], an elaborate statistical-mechanical theory, was employed. It was shown that a very large gain in the solvent entropy occurs upon protein folding. This result indicates that the translational-entropy gain of the solvent is crucially important.

We never state that the intramolecular hydrogen bonding is unimportant. It is imperative for compensating the serious penalty, the loss of hydrogen bonds with water molecules [2,15]. The formation of the α-helix leads to a great reduction of the EV for water molecules, and at the same time it ensures the intramolecular hydrogen bonds. Likewise, the formation of the β-sheet results in a considerable decrease in the EV, as well as the intramolecular hydrogen bonding. Thus, the α-helix and the β-sheet are the most advantageous unit structures. It is no wonder that these two secondary structures frequently appear in the native structure of a protein.

### 2.1. Statistical-mechanical calculation

The water-entropy gain upon protein folding can be calculated using our recently developed morphometric approach [20], combined with the angle-dependent integral equation theory [10,13,15,21–27]. We employ a multipolar water model: a water molecule is modeled as a hard sphere with diameter *d*_S_=*0.28* nm in which a point dipole and a point quadrupole of tetrahedral symmetry are embedded [21,22]. The influence of molecular polarizability of water is included by employing the self-consistent mean field (SCMF) theory [21,22]. At the SCMF level the many-body induced interactions are reduced to pairwise additive potentials involving an effective dipole moment.

Here we consider the hydration thermodynamic quantities of solutes in the isochoric (constant-volume) process. In general, the hydration energy is largely dependent on the solute-water interaction potential, while the hydration entropy is not [25,26,28]. This is particularly true for large solutes like proteins. We have calculated the hydration entropy *S_V_* (the subscript “*V*” denotes the isochoric process) for the native structures of eight peptides and proteins using the three-dimensional reference interaction site model (3D-RISM) theory combined with the all-atom potentials of the Coulomb plus Lennard-Jones (LJ) [29]. It is shown that even when the strong protein-water electrostatic interactions are completely shut off, *S_V_* changes only by ∼5%. This result is attributable to the dominant EV effect where the contribution from the water molecules near the protein is much smaller than that from those in the system. It turns out that we can choose a very simple model for the protein-water interaction potentials in calculating *S_V_*: A protein with a fixed structure is modeled as a set of fused hard spheres. The complex polyatomic structure, which is crucially important, is accounted for on the atomic level using the (*x, y, z*)-coordinates of the protein atoms as part of the input data. The diameter of each atom in the protein is set at the σ-value of the LJ potential parameters.

### 2.2. Morphometric approach

When a molecular model is employed for water, the angle-dependent integral equation theory [10,13,15,21–27] is the best one in calculating thermodynamic quantities of hydration as well as in analyzing the density and orientational structure of water near a solute. However, it is mathematically complex and cannot readily be applied to a polyatomic molecule like a protein. This problem can be solved by combining it with the morphometric approach [20].

In the morphometric approach [20], a thermodynamic quantity of hydration *Z* such as *S*_V_ is expressed using only four geometrical measures of a complex solute molecule like a protein with a fixed structure and corresponding coefficients. The expression is:

(1) $$Z=C_{1}V_{\text{ex}}+C_{2}A+C_{3}X+C_{4}Y,$$

where *V*_ex_, *A*, *X*, and *Y* are the volume excluded by the protein, the water-accessible surface area (ASA), and the integrated mean and Gaussian curvatures of the accessible surface, respectively. The idea of the morphometric form expressed by Equation (1) is that it separates the geometric properties of the solute molecule and the four coefficients. This separation allows us to determine the four coefficients in simple geometries. They are determined from calculations of *Z* for spherical solutes with various diameters followed by the application of the least square fitting. The coefficients are determined using the angle-dependent integral equation theory [10,13,15,21–27] applied to the multipolar water model [21,22]. The value of *Z* for a fixed structure of a protein is obtained only if the four geometric measures are calculated.

The sufficiently high accuracy of the morphometric approach has been demonstrated for a protein in the hard-sphere solvent [20]. It has also been verified that the accuracy is retained even when the solvent is the simple fluid in which the particles interact through strongly attractive potential like water and the particle size is as small as that of water [10]. We note that many of the hydration properties of nonpolar solutes can be reproduced even by this simple-fluid solvent [26]. It is thus reasonable to expect that the morphometric approach is sufficiently reliable for our model water as well [10].

An advantage of adopting the angle-dependent integral equation theory [10,13,15,21–27] is that the solute-water orientational correlations are explicitly taken into account and the hydration thermodynamic quantities can be decomposed into the translational and orientational contributions (i.e., contributions to the quantities from the solute-water translational correlation and from the solute-water orientational correlation, respectively). Thus, *S_V_* can be decomposed into the translational and rotational entropies of hydration: *S*_*V*,t_ and *S*_*V*,r_ (*Z* is any *of S*_*V*,t_, *S*_*V*,r_, and *S_V_*).

It is physically insightful to explore the four coefficients in Equation (1) [10]. We have found that *C*_1_ for *Z=S*_*V*,r_ is zero and hence *S*_*V*,r_ is influenced only by the ASA and curvatures of the water-accessible surface. This implies that the rotational-entropy loss is ascribed only to the water molecules in the close vicinity of the solute surface. By contrast, *S_V_*,*_t_* is strongly dependent on the EV generated by the solute because *C*_1_ takes a large, negative value. The EV-dependent term is not influenced by the surface properties.

It follows that the translational-entropy loss arises not only from the water molecules near the solute but also from those considerably far from the solute. Of course, the water molecules in the bulk (i.e., those infinitely far from the surface) cannot be influenced by the solute insertion. However, the influence reaches the length scale which is much larger than one might expect (i.e., up to the position considerably far from the solute surface). This can be understood from the following: The water molecules that are farther from the surface are less influenced, but the number of such water molecules becomes progressively larger as the distance from the surface increases. We note that in the AO theory [18,19] the EV effect is considered only in terms of the protein-water pair correlation [13,14]: The correct value of |*C*_1_| is much larger than the AO value *k*_B_*ρ*_S_.

### 2.3. Isochoric and isobaric processes

Let *ΔZ* be *Z*_N_*-Z*_U_ where the subscripts “N” and “U” represent the values for the native structure and for the unfolded state, respectively. *ΔS_V_*, for example, is the water-entropy gain upon protein folding in the isochoric process. On the other hand, the experiments are carried out in the isobaric (constant-pressure) process. The free-energy change is the same in both of the processes while the entropy-change is not. The translational-and rotational-entropy changes in the isobaric process are related to those in the isochoric process through [26]:

(2) $$\Delta S_{P,\text{t}}/k_{\text{B}}=\Delta S_{V,\text{t}}/k_{\text{B}}+(\alpha^{*}/\kappa_{T}^{*})\Delta V_{P}/d_{\text{S}}^{3},$$

(3) $$\Delta S_{P,\text{r}}/k_{\text{B}}=\Delta S_{V,\text{r}}/k_{\text{B}},$$

(4) $$\Delta H_{\text{t}}/(k_{\text{B}}T)=\Delta U_{V,\text{t}}/(k_{\text{B}}T)+(\alpha^{*}/\kappa_{T}^{*})\Delta V_{P}/d_{\text{S}}^{3},$$

(5) $$\Delta H_{\text{r}}/(k_{\text{B}}T)=\Delta U_{V,\text{r}}/(k_{\text{B}}T),$$

where *V_P_* is the partial molar volume (PMV), *H* the enthalpy, and *U* the internal energy. We note that the PMV includes the effects of the density profile of water formed near the protein while the EV does not. Δ*V_P_* is the system-volume change upon the folding in the isobaric process. The subscript “ *P* ” denotes the isobaric process. The dimensionless parameters denoted by the superscript “*”, which depend only on the properties of pure water, are defined as:

(6) $$\alpha^{*}=\alpha T,\kappa_{T}^{*}=\kappa_{T}k_{\text{B}}T/d_{\text{S}}^{3},$$

where *α* is the isobaric thermal expansion coefficient and *κ_T_* the isothermal compressibility.

The EV generated by the native structure is considerably smaller than that generated by the unfolded state. When a protein folds in the isochoric process, the EV decreases and the total volume available to water molecules in the system becomes larger. Therefore, one might think that the pressure becomes lower and hence in the isobaric process a system-volume compression occurs. However, this is not always the case [10,28]. A dense layer within which the average density of water is significantly higher than the bulk density is formed near the protein surface (the average density is almost equal to the bulk density near a hydrophobic group while it is much higher near a hydrophilic one). The folding accompanies a large decrease in the water-accessible surface area with the result that many of the water molecules forming the dense layer are released to the bulk. Due to this release, in the isochoric process the pressure does not always become lower (see 2.4).

### 2.4. Comparison between experimental and theoretical results

Terazima and coworkers elucidated the folding scheme of apoplastocyanin (apoPC) [30]. The changes of thermodynamic quantities during the folding process were measured using the transient grating (TG) and transient lens (TrL) methods in time domain at several constant temperatures. It was experimentally shown that at 298 K the folding of apoPC accompanies an enthalpic loss of 870±60 kJ/mol that is very large [10]. This result indicates that the dehydration penalty predominates over the intramolecular energy gain upon apoPC folding. It follows that the folding is driven by an even larger entropic gain.

The free-energy gain upon the folding is given by “*ΔH−T*(*ΔS_V_* +*ΔS_C_*)” where *ΔH* is the enthalpy loss and *ΔS_C_* the conformational-entropy loss. We have estimated the water-entropy gain *ΔS_V_* upon apoPC folding in the following manner [10]. First, the conformational-entropy loss *ΔS_C_* can be shown to be in the range, 305 kJ/mol<−*TΔS*_C_<956 kJ/mol. It is assumed that the free-energy gain takes the most probable value shared by a number of proteins, −50 kJ/mol. Since the enthalpic loss *ΔH* is 870 kJ/mol, the water-entropy gain is estimated to be in the range, −1876 kJ/mol<−*TΔS_V_*<−1225 kJ/mol. On the other hand, the water-entropy gain calculated using our theoretical method described above is −*TΔS_V_* = −1658 kJ/mol, which is certainly in the range estimated (the procedure of generating a set of random coils as the unfolded state is described in our earlier publication [10]). It is worthwhile to note that when the AO theory [18,19], *ΔS_V_* = −*k*_B_*ρ*_S_*ΔV*_ex_, is employed, the water-entropy gain is −*TΔS_V_* = −446 kJ/mol, which is unreasonably small. This is because the AO theory accounts for only the protein-water pair correlation component [13,14].

We have analyzed the physical origin of the large entropic gain of water [10]. The translational-entropy gain is shown to be about 20 times larger than the rotational-entropy gain. Moreover, most of the former arises from the EV effect (i.e., the term that is dependent on *V*_ex_). That is, upon the folding, the EV for water molecules in the system decreases to a large extent with the result that the restriction for the translational movement of the water molecules (i.e., the “water crowding”: see 3.1) is correspondingly reduced. The entropic gain arising from the release of water molecules in the vicinity of the protein surface to the bulk is too small to elucidate the experimental result.

A feature of apoPC folding is the extremely small difference between the unfolded state and the native structure in terms of the PMV [10]. We can consider that the folding occurs under constant-volume and constant-pressure conditions (*ΔS*_*P*,t_~*ΔS*_*V*,t_). Hence, the entropic gain originating from the EV effect is completely reflected in the experimental data. If the folding accompanies a system-volume compression (*ΔV_P_*<0), part of the entropic gain which should be reached in the isochoric process is converted to a corresponding enthalpic gain as seen from Eqs. (2) and (4) [10,28]. Such conversion does not occur in apoPC folding.

### 2.5. On the previously reported thermodynamic data for protein folding

According to the thermodynamic data available [31], for many proteins the folding accompanies negative changes in the system enthalpy and entropy. This appears to be inconsistent with our view described above and the data for apoPC folding. (There is not any reason why apoPC is an abnormal protein which should be avoided in general protein research.) Let us discuss two possible reasons for this apparent inconsistency.

The first reason is as follows. It can be shown that:

(7) $$\Delta V_{P}=\Delta V_{\text{ex}}-\xi \Delta A-A\Delta \xi ,$$

where *ξ* is a parameter representing the average density of water within the layer formed near the protein surface [12]. An exposed hydrophilic group makes a large, positive contribution to *ξ* while the contribution from an exposed hydrophobic group is essentially zero. It follows that *ξ*>1. Upon protein folding *ΔV*_ex_*<0* and *ΔA<0.* Though hydrophobic and hydrophilic groups are both buried in the folding process, the burial of hydrophobic groups is expected to be dominant, and hence *Δξ>0.* If “*ΔV*_ex_−*AΔξ*” dominates in the right hand of Equation (7), *ΔV_P_* takes a significantly large, negative value. In such cases, *ΔS_P_,*_t_ can be negative despite the fact that *ΔS*_*V*,t_ is positive (see Equation (2) and note that α*/ $\kappa_{T}^{*}$>0): A large part of the entropic gain which should occur in the isochoric process is converted to a corresponding enthalpic gain due to the system-volume compression (see Eqs. (2) and (4)), leading to negative changes in the system enthalpy and entropy [28]. It should be emphasized, however, that the translational movement of water molecules drives a protein to fold in the isobaric process as well. The second reason is in the experimental procedure itself. The experiments are usually performed for protein unfolding. When the numerical data are simply multiplied by −1, the resultant values represent the data for protein folding. Let the changes in the enthalpy, entropy, and specific heat upon the unfolding be {*ΔH*(*T*)_U_, {*ΔS*(*T*)}_U_, and {*ΔC_P_*(*T*)_U_ respectively. {*ΔH*(*T*)}_U_, for example, represents −*ΔH*(*T*). {*ΔH*(*T*)}_U_ and {*ΔC_P_*(*T*)}*U* are measured at a high temperature *T=T*_d_ where the heat denaturation occurs, and those at *T=T*_0_*=*298 K are estimated from:

(8) $$\begin{array}{c} {\left\{\Delta H(T_{0})\right\}}_{\text{U}}={\left\{\Delta H(T_{\text{d}})\right\}}_{\text{U}}-{\left\{\Delta C_{P}(T_{\text{d}})\right\}}_{\text{U}}(T_{\text{d}}-T_{0}),\\ {\left\{\Delta S(T_{0})\right\}}_{\text{U}}={\left\{\Delta H(T_{\text{d}})\right\}}_{\text{U}}/T_{\text{d}}-{\left\{\Delta C_{P}(T_{\text{d}})\right\}}_{\text{U}}\;\text{ln}\;(T_{\text{d}}/T_{0}). \end{array}$$

{*ΔH*(*T*_d_)}_U_ and {*ΔC_P_*(*T*_d_)}_U_ are both positive. {*ΔC_P_*(*T*_d_)}_U_(*T*_d_−*T*_0_) comes from the integral of {*ΔC_P_*(*T*)}_U_ from *T=T*_0_ to *T=T*_d_ where the specific-heat change is set to the value at *T=T*_d_ and treated as a constant. {*ΔC_P_*(*T*_d_)}_U_ln(*T*_d_/*T*_0_) comes from the integral of {*ΔC_P_*(*T*)}_U_/*T* from *T=T_0_* to *T=T*_d_ with the same treatment. According to our theoretical study, however, the specific-heat change largely increases as *T* becomes lower. Therefore, in Equation (8) {*ΔC_P_*(*T*_d_)}_U_(*T*_d_−*T*_0_) and {*ΔC_P_*(*T*_d_)}_U_ln(*T*_d_/*T*_0_) are seriously underestimated. It is possible that {*ΔH*(*T*_0_)}_U_ and {*ΔS*(*T*_0_)}_U_, which are actually negative, are incorrectly estimated to be positive. The experimental technique developed by Terazima *et al.* [30] enables us to directly measure *ΔH*(*T*) at *T=T*_0_*=*298 K and is free from this type of problem. It is worthwhile to re-examine the previously reported thermodynamic data using their technique.

## 3. Molecular Mechanism of Denaturation of Proteins

Pressure and cold denaturations of proteins can be elucidated consistently by our theoretical method wherein the translational entropy of water is treated as the key quantity. Heat denaturation can also be understood by considering the temperature dependence of the translational-entropy gain of water and the conformational-entropy loss of a protein molecule upon the folding.

### 3.1. Pressure denaturation

As discussed so far, the native structure with very small EV, in which the backbone and side chains are closely packed with little space in the interior, is stabilized by the water-entropy effect. One might think that the native structure is further stabilized by applying a higher pressure. However, this is not the case. It may sound strange, but pressure denaturation of proteins can certainly be elucidated in terms of the water-entropy effect.

**Figure 2.**
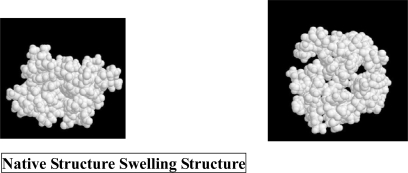
Comparison between the native structure and a swelling structure featuring the pressure-induced denaturation.

We have investigated the molecular mechanism of pressure denaturation [13] using the angle-dependent integral equation theory [10,13,15,21–27], combined with the multipole water model and the morphometric approach [20]. The hydration entropy of a protein is shown to be the key quantity. At an elevated pressure a swelling structure (see Figure 2), which has only moderately less compact than the native structure but has much larger ASA, turns more stable than the native structure in terms of the water entropy. The swelling structure is characterized by the penetration of water into the interior. In our theoretical treatment, the hydration entropy is decomposed into contributions from the restrictions of the translational and rotational freedoms of water molecules. Each contribution is further decomposed into the protein-water pair correlation component and the protein-water-water triplet and higher-order correlation component. In the latter, the effect of the so-called water reorganization (the reorganization of the water structure occurring near the solute) is also contained. The pair correlation component in the translational contribution is divided into the EV term (i.e., the term given by the AO theory) and the term arising from the water structure near the protein.

It is found that pressure denaturation accompanies a loss of the translational and rotational entropies at the protein-water pair correlation level but a much larger gain of the translational entropy at the protein-water-water triplet and higher-order correlation levels [13]. Although the translational and rotational freedoms of water molecules penetrating the protein interior and contacting the protein surface are constrained, the translational restriction for the water molecules well outside the protein is greatly reduced. The latter entropic gain dominates, leading to the denaturation.

The above result can be interpreted as follows [13]. An important clue is the “water crowding”. The presence of a water molecule generates an EV for the other water molecules in the system, hindering their translational movement. This crowding effect is quite serious at high pressures. Although the movement of the water molecules contacting the surface and penetrating into the interior is largely restricted, the EVs generated by these water molecules as well as by the protein overlap one another due to the contact and penetration. As a consequence, the restriction of the translational movement of the other water molecules in the system is greatly reduced, leading to a gain in the total entropy. This water-entropy gain is ascribed to the protein-water-water triplet and higher-order correlations. In other words, the contact and penetration originate from the triplet and higher-order correlation effect. If the attention was paid to the pair correlation alone as in the AO theory, the conclusion would be that the native structure was further stabilized relative to the swelling structure by applying a higher pressure.

In the morphometric form of Equation (1) applied to *Z=S_V_* at the ambient pressure, *C*_1_<0, *C*_2_>0, and |*C*_1_| ≫*C*_2_ [13]. Hence, *S_V_* is determined mainly by the EV term. As the pressure becomes higher, both |*C*_1_| and *C*_2_ increase. However, the increase in *C*_2_ is considerably larger: it is over an order of magnitude larger at a high pressure where the denaturation occurs than at the ambient pressure [13]. At the high pressure the ASA term as well as the EV term is important. The positive sign of *C*_2_ stems from the protein-water-water triplet and higher-order correlation effect. The pressure dependence of the structural stability is determined by a subtle balance between the two terms in the morphometric form, *C*_1_{*ΔV*_ex_}_U_ and *C*_2_{*ΔA*}_U_. {*ΔV*_ex_}_U_ and {*ΔA*}_U_ are both positive. At high pressures unfolded structures not only with *ΔA* that is as large as possible but also with *ΔV*_ex_ kept sufficiently small are stabilized ({*ΔS_V_* }_U_>0). A transition to a random-coil state undergoes an unacceptably large increase in the EV though the ASA is greatly enlarged. Water penetration into the interior is a good solution leading to much larger ASA and only moderately larger EV. Thus, a structure featuring pressure denaturation is characterized by the swelling, water penetration into the interior, and only a moderate reduction of the compactness. This result is in qualitatively good agreement with the experimental observations [11–14].

Experimental studies have shown that the amyloid-fibril formation [32], association of virus [33], and formation of actin filaments [34] are also entropically driven. By applying a high pressure, the fibrils, filaments, and virus are dissociated. These phenomena are closely related to pressure denaturation of proteins. We have recently developed a general theoretical framework [14] of pressure effects on the structures formed by the self-assembly of solute molecules immersed in solvent. The basic physics is in the phenomenon that when a large hard-sphere solute is immersed in small hard spheres forming the solvent, the small hard spheres are enriched near the solute and this enrichment becomes greater as the pressure increases. “Attractive interaction” is entropically induced between the solute surface and solvent particles, and many solvent particles are driven to contact the solute surface. The attractive interaction becomes higher with rising pressure. The formation of the enriched layer itself causes an entropic loss because the translational movement of solvent particles within the layer, in particular, that of those in contact with the solute surface, is somewhat restricted: An entropic loss occurs at the solute-solvent pair correlation level. However, the solvent crowding (i.e., the restriction of the translational movement of a solvent molecule by the other solvent molecules) well outside the layer is largely reduced: An entropic gain occurs at the solute-solvent-solvent triplet and higher-order correlation levels. The attractive interaction induced between the solute surface and solvent particles originates from the triplet and higher-order correlations. The key quantity is the net solvent-entropy gain arising from the loss at the pair correlation level and the gain at the triplet and higher-order correlation levels. The density profile of solvent particles around the solute is determined so that the key quantity can be maximized. These results are applicable to a complex solute whose structure is changeable using the morphometric approach [14]. The structure stabilized, which maximizes the key quantity, is dependent on the pressure. The structure stabilized at high pressures is quite different from that at low pressures. Though at low pressures a structure almost minimizing its EV is stabilized, at high pressures a structure with the largest possible ASA together with the EV kept sufficiently small is more favored.

### 3.2. Cold denaturation

Our recent development on the molecular mechanism of cold denaturation [15] is briefly described here. The temperature dependence of the hydrophobicity of nonpolar groups provides an important clue to the molecular mechanism. We have analyzed the terms in *ΔS_V_* and *ΔU_V_* which are determined by the EV and by the ASA and the surface curvature (SC), respectively. At low temperatures, the ordered structure with enhanced hydrogen bonds of water molecules is formed near nonpolar groups (such enhancement does not occur at ambient temperature [26]). The enhancement becomes more important for unfolded structures with larger ASA. At low temperatures, the unfolded structures are relatively more destabilized in respect of the ASA- and SC-dependent term of *ΔS_V_* but more stabilized in respect of the ASA- and SC-dependent term of *ΔU_V_*. Interestingly, the destabilization and the stabilization, which are both quite large, are almost cancelled out: The formation of the ordered structure itself cannot be the driving force in cold denaturation.

At low temperatures, both the native structure and unfolded structures are less hydrophobic in the sense that *μ/*(k_B_T) (*μ* is the hydration free energy) is significantly reduced (it should be noted that *μ* is even more reduced). However, the reduction is greater for a structure with larger EV, and the EV-dependent term in *ΔS_V_* plays an essential role. The EV-dependent term can further be decomposed into the protein-water pair correlation (i.e., the AO) component and the protein-water-water triplet and higher-order correlation component. We have found that the latter component is responsible for the reduction: The translational-entropy effect arising from the water in the system, by which the native structure is stabilized relative to the unfolded structures, is considerably less powerful when the temperature is lowered, leading to cold denaturation. As in the case of pressure denaturation, the AO theory is incapable of elucidating cold denaturation.

### 3.3. Heat denaturation

The enthalpy change upon protein folding decreases as *T* becomes higher (*dΔH/dT<*0)*,* and *ΔH* takes a negative value at *T*=*T*_m_ (*T*_m_ is the denaturation temperature). Therefore, thermal denaturation cannot be driven by an enthalpic gain. Since the change in the protein intramolecular energy is independent of *T*, the change in the hydration enthalpy decreases as *T* becomes higher. The changes in the hydration enthalpy and *ΔS*_V_ are somewhat compensating [31], and *ΔS*_V_ is a decreasing function of *T*. This has been verified by our theoretical study [15].

It has been verified by experiments [35] that the loss of the conformational entropy (CE) upon protein folding increases as *T* becomes higher. The CE is closely related to the allowed ranges of dihedral angles which are dependent on the torsion energy and *T*. Angles giving high torsion energy are not allowed at a low temperature. As *T* increases, the allowed range of each angle becomes increasingly wider, leading to larger CE of the denatured state. The CE of the native structure does not become significantly larger due to the constraints caused by the closely packed structure. It follows that |*ΔS_C_*| (*ΔS_C_*<0 is the CE change upon the folding) becomes larger with increasing *T*. There is the experimental evidence that the change in the specific heat upon protein folding is negative [30]. The temperature dependences of *ΔH, ΔS*_VH_, and *ΔS*_C_ discussed above are consistent with the evidence.

As *T* becomes higher *ΔS*_V_ decreases while |*ΔS*_C_| increases, and this effect should be a major cause of thermal denaturation. We have recently proposed a physical picture of thermal denaturation of proteins. In the picture, the structural stability is determined by the competition of the water-entropy gain upon the folding per residue and the conformational-entropy loss per residue. An important finding is that the water-entropy gain evaluated at 298 K divided by the number of residues is a good measure of the thermal stability. A protein with a larger value of this measure tends to have higher *T*_m_. More details will be published in a future article [36].

## 4. Novel Method for Predicting the Native Structure

Predicting the native structure of a protein from its amino-acid sequence is one of the most challenging subjects in molecular biology, biophysics, and biochemistry. As the first step, the development of a free-energy function which takes the minimum value for the native structure is highly desired. However, there are two major stumbling blocks to overcome. First, the complex effects of water must reasonably be taken into account in the free-energy function. Secondly, the calculation of the function is to be accomplished quite rapidly because the number of candidate structures is enormous. Here we briefly describe our ongoing efforts toward the development of a reliable method for predicting the native structure of a protein.

### 4.1. Free-energy function based on all-atom model for a protein

We have developed a simple, new free-energy function *F* which is crucially important in arguing the structural stability of a protein [17]:

(9) $$F=-TS_{V}+\Lambda ,$$

where *S_V_* is the hydration entropy in the isochoric process and calculated using the angle-dependent integral equation theory [10,13,15,21–27] combined with the multipolar water model [21,22] and the morphometric approach [20]. *Λ* corresponds to the sum of the hydration energy (not free energy) and the intramolecular energy when the fully extended structure is chosen as the standard one with *Λ=*0. *Λ* is the total dehydration penalty accompanying a transition to a more compact structure. Compared to the fully extended structure which possesses the maximum number of hydrogen bonds with water molecules and no intramolecular hydrogen bonds, in a more compact structure some donors and acceptors (e.g., N and O, respectively) are buried in the interior after the break of hydrogen bonds with water molecules (CO...W; NH...W, etc.), leading to a positive value of *Λ*. There is no problem if the intramolecular hydrogen bonds (CO...HN, etc.) are formed. However, such hydrogen bonds are not always formed, leading to the dehydration penalty.

When a donor and an acceptor are buried in the interior after the break of hydrogen bonds with water molecules, if they form an intramolecular hydrogen bond, we impose no penalty. On the other hand, when a donor or an acceptor is buried with no intramolecular hydrogen bond formed, we impose the penalty of *λ* [17]. We have to determine if each of the donors and acceptors is buried or not. The water-accessible surface area is calculated for each of them. If it is zero, the donor or acceptor is considered buried. That is, the dehydration penalty of *λ* is imposed only when the donor or acceptor is completely buried. We examine all donors and acceptors for backbone-backbone, backbone-side chain, and side chain-side chain intramolecular hydrogen bonds and calculate *Λ*. *−S_V_* and *Λ* are both positive and strongly dependent on the protein structure. Currently, *λ* is set at 17.4 kJ/mol at 298 K: this value is based on the results from the computer simulation performed by Brooks *et al.* [37] for hydrogen-bond formation between two formamide molecules in a nonpolar liquid and in water (the details are described in our earlier publication [17]). A more reliable value of *λ* is to be determined in a future study.

### 4.2. Discrimination of the native fold from misfolded decoys

**Figure 3.**
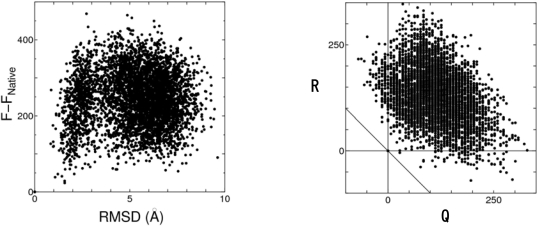
Left: *F*–*F*_Native_ plotted against the RMSD from the native structure for all the seven proteins in the 4state_reduced decoy set. Right: *R* plotted against *Q* for all the seven proteins in the 4state_reduced decoy set. The three straight lines drawn represent *Q*=0, *R=0,* and *Q+R=0,* respectively.

We have tested the function defined by Equation (9) for “decoy” sets of many different proteins (a total of fifty proteins chosen from the 4state_reduced, fisa, fisa_casp3, and rosetta decoy sets – we will test more in the near future). The (*x*, *y, z*)-coordinates of the protein atoms are used as part of the input data to characterize each structure. All the unreasonable overlaps of the protein atoms are eliminated using a standard energy-minimization method with the all-atom potentials of the Coulomb plus LJ [17]. It is demonstrated that the native fold can be discriminated from the misfolded decoys with 100% accuracy (the result for the 4state_reduced decoy set is shown in Figure 3). The free-energy function is better than any other physics-based or knowledge-based potential function in terms of the accuracy and the achievement of high *Z*-scores. Even when *λ* is increased or decreased by ∼50%, for example, the result is successful in the sense that the free-energy function becomes the lowest for the native structure, which indicates the robustness of our method against the uncertainty of *λ* (more details will be published in a future article [38]).

We define *Q* and *R* as:

(10) $$Q=\Lambda -\Lambda_{\text{Native},}\;R=-TS_{V}-{\left\{-TS_{V}\right\}}_{\text{Native},\;}\;Q+R=F-F_{\text{Native}},$$

where the subscript “Native” denotes the value for the native structure. *Q* and *R* share almost the same magnitude (see Figure 3). On the whole, *Q* and *R* are anticorrelated for the following reason. When a protein takes a more compact structure, for example, the hydration entropy usually becomes smaller. At the same time the burial of “CO” and “NH” occurs, but it is not always possible to completely make up for the break of hydrogen bonds with water molecules, giving rise to a larger value of *Λ*. There are significantly many structures giving *R<0.* There are even more structures with *Q<0.* However, there is no structure causing *Q*+*R<0*. The structures giving higher entropy of water than the native structure can certainly be constructed. However, such structures suffer seriously more total dehydration penalty. The structures undergoing only less total dehydration penalty than the native structure can also be constructed readily, but such structures give rise to seriously lower entropy of water. The native structure is optimized in terms of the sum of the two important factors [17].

### 4.3. Toward development of a reliable tool for predicting the native structure of proteins

No large computer memory is required in our method and the calculation of *F* is finished in about 0.1 sec for one structure on our workstation. Another great advantage is that we can avoid the use of the protein force-field parameters which are more or less uncertain. We note that a simple method capturing the essential physics is often more powerful than a computer simulation incorporating all the details. In the latter, the results obtained are strongly dependent on the force-field parameters used [39].

Our free-energy function is best suited to selecting the most stable structure from among the candidate structures. The number of the candidate structures is allowed to be huge, because in our method the function is calculated with minor computational effort. It may be possible to develop a practical tool for predicting the native structure of a protein from its amino-acid sequence, by combining our method with the bioinformatics techniques which can generate a variety of candidate structures. Our method is capable of handling very large proteins and can also be extended to analyses of the protein-protein interaction and protein aggregation.

## 5. Conclusions

We have shown that the entropic effect arising from the translational movement of water molecules is a principal driving force in protein folding. This is true in a variety of other self-assembly and ordering processes in biological systems, such as molecular recognition [3–5] and ordered aggregation of protein molecules [6,7]. Based on this view, we have succeeded in elucidating the microscopic mechanisms of protein folding [1,2,10] and denaturation [11–15,36], receptor-ligand binding [3–5], and amyloid-fibril formation [6,7]. By “translational movement of water molecules” we do not refer to dynamical aspects of the behavior of the water-protein system. What we claim is that upon protein folding, for example, the number of accessible translational configurations of water that coexists with the protein increases to a large extent.

The solvent-entropy effect becomes larger as the solvent number density increases and/or the molecular size decreases [1,2]. Thanks to the hydrogen bonding, water can exist in the liquid (dense fluid) state at ambient temperature and pressure despite its exceptionally small molecular size. The effect is the largest for water among all ordinary liquids in nature (in several studies [40–44], the small molecular size has been pointed out as an important feature of water causing the hydrophobic effect). We can thus understand why water is indispensable to life through the self-assembly and ordering processes.

The change in the water-entropy upon protein folding/unfolding can be decomposed into the translational and orientational contributions [10,26]. The translational contribution can be decomposed into the EV-dependent term and the ASA- and SC-dependent term. They can further be decomposed into the protein-water pair and protein-water-water triplet and higher-order correlation components [13,14,26]. At low pressures, the triplet and higher-order correlation component of the EV-dependent term in the translational contribution is dominant. If this component is overlooked, the large entropic gain observed for apoPC folding [10] and cold denaturation [15] cannot be explained. At high pressures, the triplet and higher-order correlation component of the ASA- and SC-dependent term in the translational contribution becomes equally important. Only by accounting for this component, pressure denaturation [11–14] can reasonably be elucidated.

In light of the successful results in elucidating the folding/unfolding mechanisms, we have developed a novel free-energy function for a protein. We intend to further improve the function and construct a reliable method for predicting the native structure of a protein from its amino-acid sequence. It is worthwhile to measure the enthalpy change upon the folding at prescribed temperatures for proteins using the experimental technique developed by Terazima *et al.* [30]. The system-volume change is also an important quantity to be measured. A major concern is to compare the data with those which are already available. A long review on the crucial importance of the translational movement of water molecules in sustaining life was given in our earlier publication [45].
